# *In vitro* and *in vivo* anti-tumor activity of alectinib in tumor cells with NCOA4-RET

**DOI:** 10.18632/oncotarget.17900

**Published:** 2017-05-16

**Authors:** Sachiko Arai, Kenji Kita, Azusa Tanimoto, Shinji Takeuchi, Koji Fukuda, Hiroshi Sato, Seiji Yano

**Affiliations:** ^1^ Division of Medical Oncology, Cancer Research Institute, Kanazawa University, Kanazawa, Japan; ^2^ Division of Molecular Virology and Oncology, Cancer Research Institute, Kanazawa University, Kanazawa, Japan

**Keywords:** RET fusion, lung cancer, alectinib, pleural carcinomatosis, NCOA4-RET

## Abstract

Rearranged during transfection (RET) fusion-positive non-small cell lung cancer (NSCLC) accounts for approximately 1–2% of all NSCLCs. To date, RET fusions that involve at least six fusion partners in NSCLC, such as KIF5B, CCDC6, NCOA4, TRIM33, CLIP1, and ERC1, have been identified. Recent clinical trials for RET fusion-positive NSCLC using vandetanib or cabozantinib demonstrated positive clinical response and considerable differential activities for RET inhibitors among fusion partners. Alectinib, an approved ALK inhibitor, is reported to inhibit KIF5B-RET and CCDC6-RET. However, the activity of alectinib with respect to RET with other fusion partners is unknown. In the present study, we investigated the effects of alectinib on NCOA4-RET fusion-positive tumor cells *in vitro* and *in vivo*. Alectinib inhibited the viability of NCOA4-RET-positive EHMES-10 cells, as well as CCDC6-RET-positive LC-2/ad and TPC-1 cells. This was achieved via inhibition of the phosphorylation of RET and induction of apoptosis. Moreover, alectinib suppressed the production of thoracic tumors and pleural effusions in an orthotopic intrathoracic inoculation model of EHMES-10 cells. *In vivo* imaging of an orthotopically inoculated EHMES-10 cell model also revealed that alectinib could rescue pleural carcinomatosis. These results suggest that alectinib may be a promising RET inhibitor against tumors positive for not only KIF5B-RET and CCDC6-RET, but also NCOA4-RET.

## INTRODUCTION

The rearranged during transfection (RET) gene was discovered in 1985 as an oncogene produced by recombination during the transfection of NIH 3T3 cells with human lymphoma DNA [1]. RET is located at 10q11.2, which encodes RET receptor tyrosine kinase. This gene plays important physiological roles in neural and renal development [2]. RET fusion genes are thought to be oncogenic drivers and they are detected in 20–40% of all papillary thyroid cancers [3]. The gene product of the RET fusion gene forms a dimer with the receptor tyrosine kinase, causing constitutive kinase activation [4].

RET fusion genes were recently identified in a population of non-small cell lung cancers (NSCLCs) [5–8]. The most common (>80%) fusion partner for RET is KIF5B, followed by CCDC6, NCOA4, TRIM33, CLIP1, and ERC1 [9, 10]. RET fusion is detected in 1–2% of all NSCLCs, and it is mutually exclusive to the mutation or rearrangement of other genes, including EGFR, KRAS, ALK, and ERBB2 [9]. The KIF5B-RET transgenic mice develop lung adenocarcinomas [11, 12], indicating that RET fusion genes are oncogenic drivers of lung adenocarcinomas. Recent clinical trials for RET fusion-positive NSCLCs using vandetanib or cabozantinib demonstrated good clinical responses [10, 13]. Vandetanib showed a response rate of 53% (9/17 cases). Interestingly, vandetanib showed much higher response rates in CCDC6-RET-positive NSCLCs (83% [5/6 cases]) compared to KIF5B-RET-positive NSCLCs (20% [2/10 cases]) [13]. Cabozantinib showed a response rate of 28% (7/25 cases). Cabozantinib showed a response to KIF5B-RET (20% [3/15 cases]), unknown genes with FISH-positive (33% [2/3 cases]), TRIM33-RET, and CLIP1-RET, but not CCDC6-RET or ERC1-RET [10]. These findings suggest that RET fusion-positive NSCLCs with different fusion partners may have variable susceptibilities to different RET tyrosine kinase inhibitors (TKIs).

Alectinib is a second generation ALK-TKI approved for ALK-rearranged NSCLC [14]. Although alectinib is thought to be a very selective inhibitor for ALK compared to other compounds that are approved for ALK-positive NSCLCs, such as crizotinib and ceritinib, alectinib also has high activity against RET [15]. We are now conducting a clinical trial to assess the efficacy of alectinib in RET-positive NSCLCs (ALL-RET study, UMIN000010095) [16]. Alectinib is reported to have inhibitory activity against KIF5B-RET and CCDC6-RET [15], but it is unknown whether alectinib has activity for other types of RET fusions.

In the present study, we investigated the effect of alectinib on NCOA4-RET utilizing native tumor cells with this type of RET fusion. We compared this to tumor cells harboring CCDC6-RET, which were shown to have sensitivity to vandetanib in an earlier clinical trial [13].

## RESULTS

### Alectinib inhibits the viability of tumor cells with NCOA4-RET

**Figure 1:**
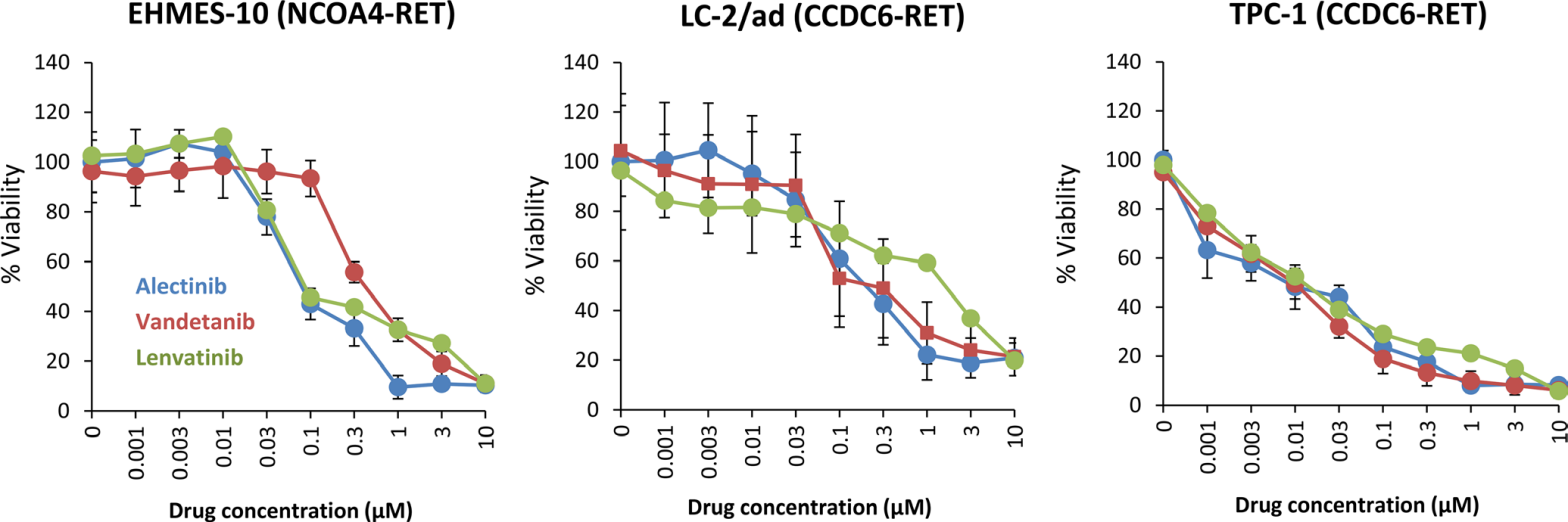
Alectinib reduces the viability of tumor cells with NCOA4-RET *in vitro* Tumor cells (2 × 10^3^ cells/well) were incubated with various concentrations of alectinib, vandetanib, or lenvatinib for 72 hours. Cell viability was determined using the MTT assay. Bars represent SD of quadruplicate cultures. Data shown are representative of three independent experiments yielding similar results.

The mesothelioma cell line EHMES-10 [17–19] containing a NCOA4-RET fusion, and human lung adenocarcinoma cell line LC-2/ad [20] and thyroid papillary carcinoma cell line TPC-1 [21] containing a CCDC6-RET fusion were used in this study. In the first set of experiments, we examined the effect of alectinib by comparing it with the other RET kinase inhibitors, vandetanib and lenvatinib. We examined the viability of RET-fusion-positive human tumor cell lines. Alectinib inhibited the viability of NCOA4-RET-positive EHMES-10 cells and CCDC6-RET-positive LC-2/ad and TPC-1 cells in a dose-dependent manner. Vandetanib and lenvatinib also inhibited the viability of these three cell lines in a dose-dependent manner, although there were minor differences in its efficacy among the cell lines (Figure 1).

### Alectinib inhibits the phosphorylation of NCOA4-RET protein

**Figure 2:**
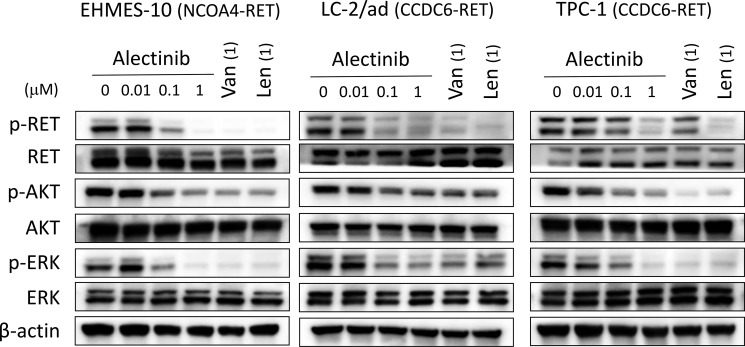
Alectinib inhibits the phosphorylation of NCOA4-RET protein *in vitro* Tumor cells were incubated with the indicated concentrations of alectinib, vandetanib (Van), or lenvatinib (Len) for 2 hours. Cell lysates were obtained and analyzed by immunoblotting with antibodies against the indicated proteins. Data shown are representative of three independent experiments yielding similar results.

We examined the effect of phosphorylation of RET and its downstream targets, ERK and AKT. Western blot analyses revealed that alectinib inhibited the phosphorylation of RET, ERK, and AKT in NCOA4-RET-positive EHMES-10 cells and CCDC6-RET-positive LC-2/ad and TPC-1 cells (Figure 2). Vandetanib and lenvatinib at concentrations of 1 µM also inhibited the phosphorylation of RET, ERK, and AKT in all three cell lines. These results suggest that alectinib, like vandetanib and lenvatinib, suppressed the viability of tumor cells harboring NCOA4-RET and CCDC6-RET by inhibiting RET activity and its downstream pathways.

### Alectinib induces apoptosis in tumor cells possessing NCOA4-RET

**Figure 3:**
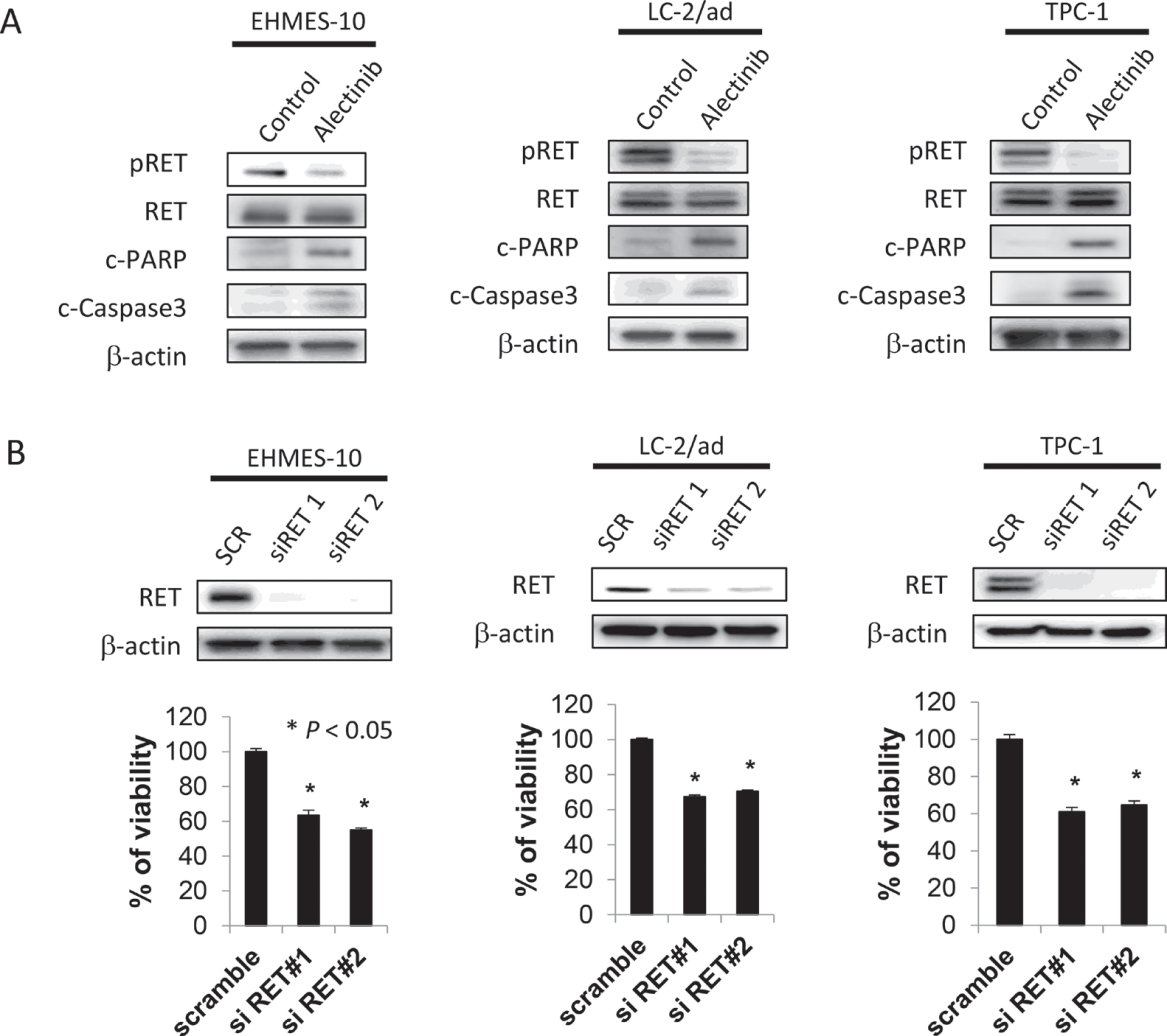
Alectinib induces apoptosis of EHMES-10 cells with NCOA4-RET *in vitro* (**A**) Tumor cells (2 × 10^5^ cells/well) were treated with alectinib for 48 hours. (**B**) Tumor cells (2 × 10^5^ cells/well) were treated with scramble siRNA or RET specific siRNA for 72 hours. Cell lysates were obtained and analyzed by immunoblot with antibodies toward the indicated molecules. Data shown are representative of three independent experiments yielding similar results.

We next examined whether alectinib induced apoptosis in tumor cells with RET fusion genes. Treatment with alectinib for 48 h inhibited RET phosphorylation and induced expression of apoptotic markers (cleaved PARP and cleaved caspase-3) in NCOA4-RET-positive EHMES-10 cells. This was also observed in CCDC6-RET-positive LC-2/ad and TPC-1 cells (Figure 3A). Moreover, treatment with RET-specific siRNAs successfully knocked down RET protein expression and reduced the viability of all three cell lines tested (Figure 3B). These results indicate that alectinib induces apoptosis in not only CCDC6-RET-positive cells, but also in NCOA4-RET-positive cells.

### Alectinib rescues the pleural carcinomatosis produced by NCOA4-RET-positive tumor cells

**Figure 4:**
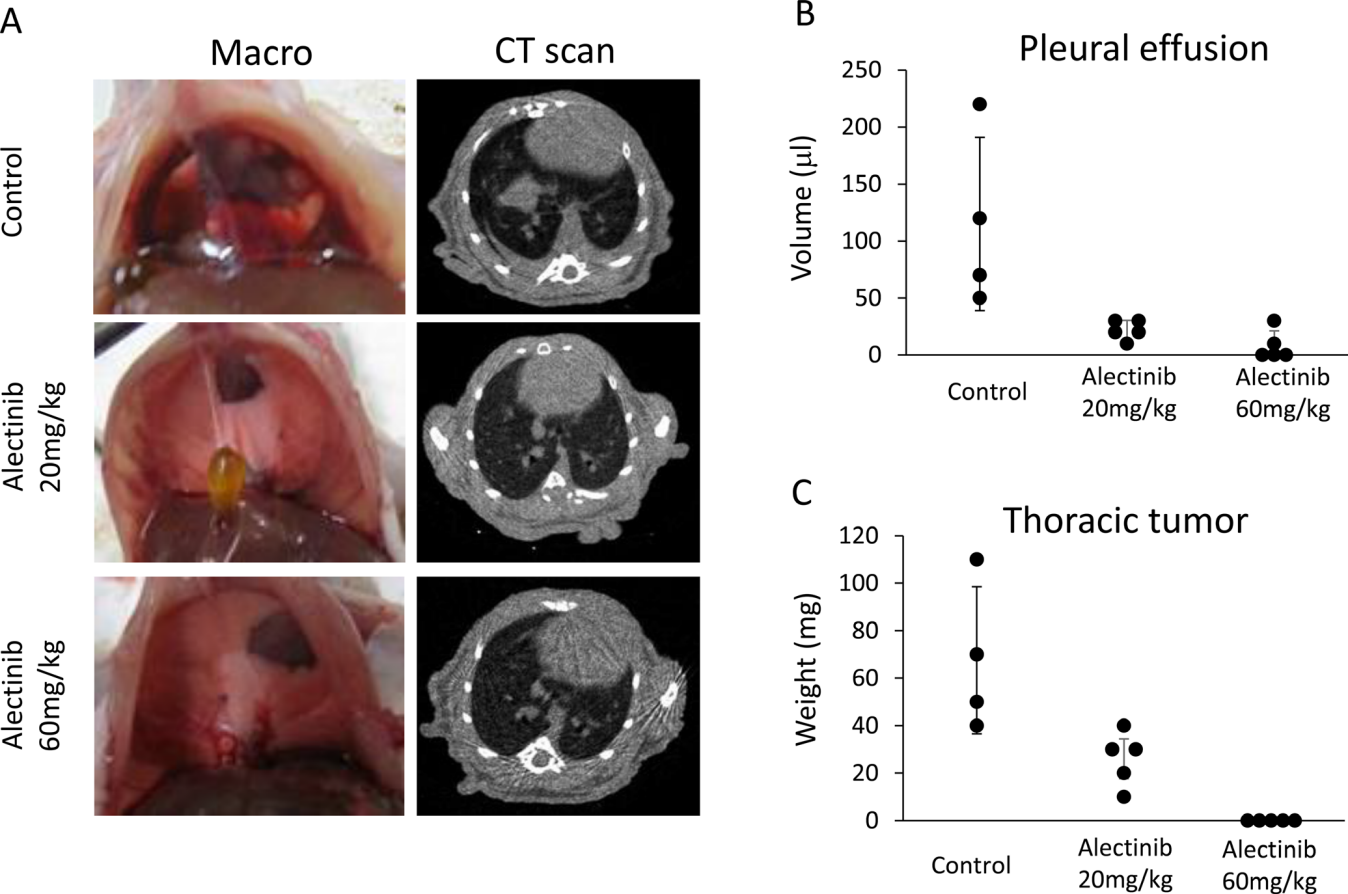
Alectinib inhibits the production of intrathoracic lesions and pleural effusions by tumor cells with NCOA4-RET EHMES-10 cells (1 × 10^6^) were inoculated into the thoracic cavities of SHO-SCID mice (*N* = 14). The mice were treated with control (*N* = 4) or alectinib at a concentration of 20 mg/kg (*N* = 5) or 60 mg/kg (*N* = 5) daily from day 14 to day 28. On day 28, CT scans were performed to evaluate the production of pleural effusions and thoracic tumors (**A**). Mice were then sacrificed and images of the thoracic cavity were obtained. Pleural effusions (**B**) and thoracic tumors (**C**) were measured. Dots show the values of individual mouse. Bars show means ± SD.

**Figure 5:**
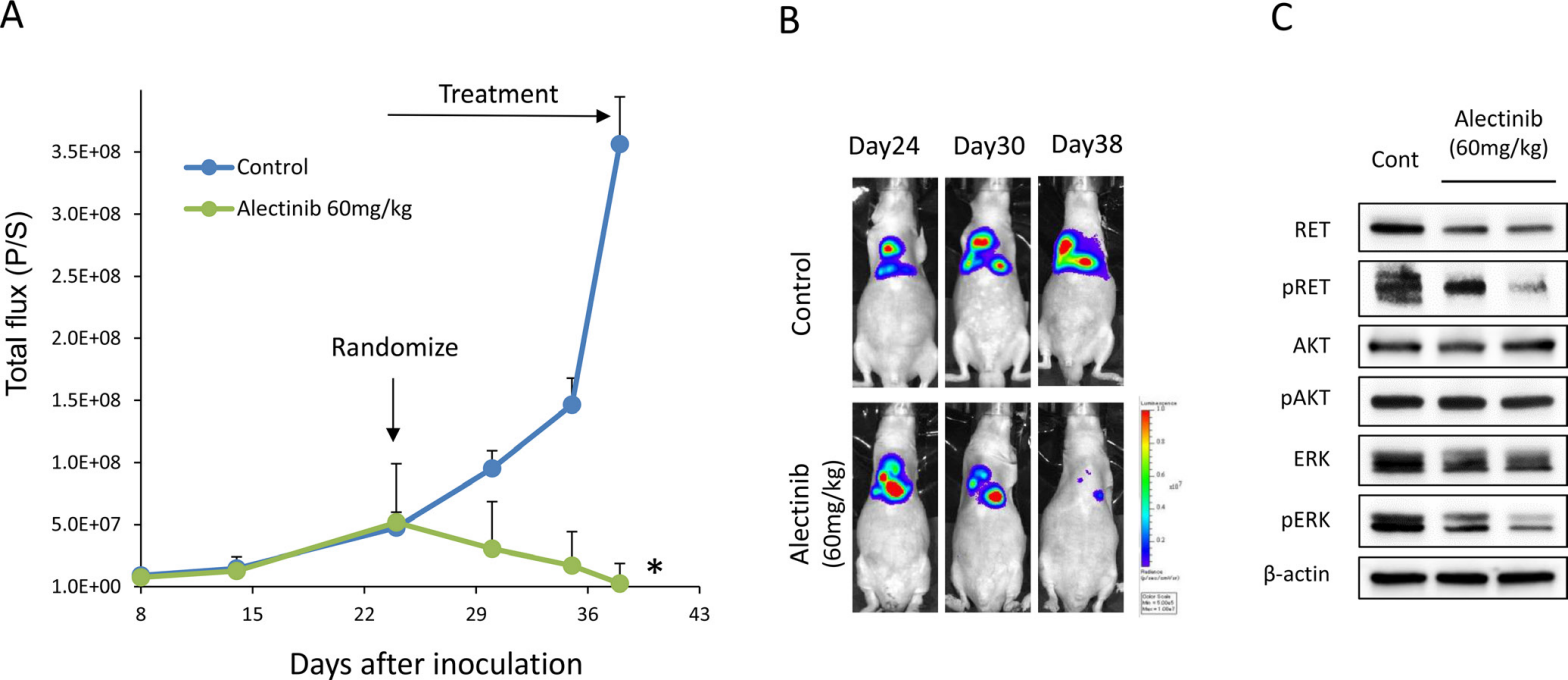
Alectinib delays the intrathoracic progression of tumor cells with NCOA4-RET (**A**) EHMES-10/Eluc cells (1 × 10^6^) were inoculated into the thoracic cavities of SHO-SCID mice (*N* = 10). The mice were treated with control (*N* = 5) or alectinib at a concentration of 60 mg/kg (*N* = 5) daily from day 24 to day 38. Bioluminescence was measured twice a week by IVIS. Data are the means ± SE. *p < 0.05 compared to control group. (**B**) Representative images are shown. (**C**) On day 38, mice were sacrificed and thoracic tumors were harvested. Cell lysates were obtained and analyzed by immunoblotting with antibodies against the indicated proteins.

We further assessed the effect of alectinib *in vivo*. As we previously reported [17, 18], EHMES-10 cells developed pleural carcinomatosis accompanied by thoracic tumors and malignant pleural effusions within 28 days after intrathoracic (orthotopic) inoculation (Figure 4A). Alectinib treatment given from day 15 to day 28 remarkably inhibited the production of thoracic tumors and pleural effusions as determined by CT scans on day 28 (Figure 4A). Accordingly, the weight of thoracic tumors and the volume of pleural effusions in alectinib-treated mice were significantly lower than those of control mice (Figure 4B, 4C). To further assess whether alectinib rescued pleural carcinomatosis, we utilized an *in vivo* imaging model. For this model, we established luciferase-transfected EHMES-10 cells (EHMES-10/Eluc). EHMES-10/Eluc cells had a similar sensitivity to alectinib and vandetanib when compared to the parental EHMES-10 cells *in vitro* (Supplementary Figure 1). We detected elevated bioluminescence in mice inoculated with EHMES-10/Eluc cells by day 24, indicating the presence of pleural carcinomatosis. Bioluminescence in control mice consistently increased over the course of the experiment, but bioluminescence in alectinib-treated mice decreased. These results clearly indicated that alectinib treatment rescued the pleural carcinomatosis produced by EHMES-10/Eluc cells (Figure 5A, 5B). Continuous treatment with alectinib at 60 mg/kg/day did not cause body weight loss in the mice (Supplementary Figure 2). We harvested the thoracic tumors of these mice and assessed the extent of RET phosphorylation by western blot. Although there were individual differences, alectinib treatment tended to inhibit phosphorylation of RET and ERK in thoracic tumors (Figure 5C). These results indicate that alectinib can rescue the pleural carcinomatosis produce by NCOA4-RET-positive tumor cells *in vivo,* likely via inhibition of RET phosphorylation.

## DISCUSSION

In the present study, we demonstrated that alectinib is effective at inhibiting native tumor cell lines harboring NCOA4-RET (EHMES-10). In addition, we established an orthotopic *in vivo* imaging model of EHMES-10 cells, and demonstrated the anti-tumor efficacy of alectinib in this model using bioluminescence and CT scans. We also measured tumor weights and pleural effusion volumes. Alectinib treatment could rescue the pleural carcinomatosis caused by the EHMES-10 cells. These findings suggest that alectinib may be useful in cancer patients who are positive for NCOA4-RET and CCDC6-RET.

EHMES-10 is a unique cell line. It was established from the pleural effusion of a malignant mesothelioma patient [19]. EHMES-10 cells were known to produce high amounts of VEGF and develop massive bloody pleural effusions, mimicking clinical features of pleural mesotheliomas when inoculated orthotopically into the pleural cavities of immune-deficient mice [17]. In previous studies, we found that this cell line had NCOA4-RET, also called RET/PTC3. Additionally, vandetanib treatment significantly inhibited the production of thoracic tumors and pleural effusions in the orthotopic model [18]. This observation was confirmed in this study using the *in vivo* imaging model with EHMES-10/Eluc cells (Supplementary Figure 3). More importantly, alectinib rescued pleural carcinomatoses in this model. The clinical efficacy of alectinib in RET-positive NSCLCs is under evaluation [16]. Our preclinical results support the enrollment of NCOA4-RET-positive NSCLC patients in a clinical trial of alectinib.

It is interesting to note that while alectinib inhibited phosphorylation of both ERK and AKT in EHMES-10 cells *in vitro* (Figure 2), alectinib treatment resulted in inhibition of only ERK phosphorylation *in vivo* (Figure 5C). The reason of this discrepancy is unknown at present. In the *in vitro* culture of EHMES-10 cells, ERK phosphorylation seems to be suppressed more efficiently than AKT phosphorylation by alectinib (Figure 2). Relatively low concentration of alectinib may penetrate orthotopic tumors, and therefore only ERK phosphorylation, but not AKT phosphorylation, might be inhibited *in vivo*.

The NCOA4-RET fusion gene is detectable in several types of cancer, including thyroid papillary carcinoma [22–24], NSCLC [25], colon cancer [26, 27], and salivary gland duct cancer [28]. However, the incidence is generally rare. Recent studies reported that NCOA4-RET was detected in 1/936 NSCLCs [25], 1/3117 colon cancers [27], and 2/149 salivary gland cancers [28]. On the other hand, the incidence of NCOA4-RET in thyroid papillary carcinomas varies (1–86.7%) among reports. Rao et al. reported that the prevalence of NCOA4-RET (RET/PTC3) in thyroid papillary carcinomas in the Chennai population was 86.7% [22]. Ushenkova et al. reported that NCOA4-RET (RET/PTC3) was detected in 23.6% of all radiogenic thyroid papillary carcinomas in children affected after the Chernobyl accident [23]. These results suggest that the incidence of NCOA4-RET (RET/PTC3) may vary among different ethnicities, generations (age), and causes of cancer (sporadic or radiogenic). Analyses in larger cohorts are warranted to clarify the precise prevalence and characteristics of thyroid papillary carcinomas that present with NCOA4-RET (RET/PTC3).

RET-fusion genes are mutually exclusive with respect to their presence in NSCLCs compared to other driver oncogenes, such as EGFR-activating mutations and ALK translocations [9]. However, RET-fusion genes were incidentally detected in tumors that possessed other driver oncogenes after the acquisition of resistance to corresponding targeted drugs. Klempner et al. reported three EGFR-mutated NSCLC patients with RET-fusion genes that were concomitantly detected with the original EGFR mutation after acquisition of resistance to EGFR TKIs (two cases with CCDC6-RET and one case with NCOA4-RET) [24]. These reports suggest that RET-fusion genes, including NCOA4-RET, could act as an alternative driver in tumors when the original driver signaling is blocked.

KIF5B is the most prevalent (>80%) fusion partner of RET-fusion in NSCLCs [9]. In the experiments with BaF3 and NIH-3T3 cells transfected with RET-fusion genes, cells with KIF5B-RET were shown to have similar sensitivity to vandetanib and lenvatinib when compared to those with CCDC6-RET and NCOA4-RET [15, 29, 30]. However, clinical trials with RET inhibitors, including vandetanib, suggested that there is a considerable difference in the efficacy of RET inhibitors among RET-fusion partners. This indicates a limitation of experiments that involve only BaF3 and NIH-3T3 cells transfected with RET fusion genes. Unfortunately, native tumor cell lines with KIF5B-RET are not available or reported to our best knowledge. Therefore, KIF5B-RET-positive tumor cell lines need to be established from clinical specimens to obtain more clinically relevant findings. Establishment of native tumor cell lines with other RET-fusions, including TRIM33-RET, CLIP1-RET, and ERC1-RET, is also warranted.

In conclusion, we demonstrated that alectinib has inhibitory effects on tumor cells with NCOA4-RET *in vitro* and *in vivo*. Our preclinical results support the enrollment of NCOA4-RET-positive cancer patients in a clinical trial of alectinib.

## MATERIALS AND METHODS

### Cell cultures and reagents

The mesothelioma cell line EHMES-10 [17–19] containing a NCOA4-RET fusion, and human lung adenocarcinoma cell line LC-2/ad [20] and thyroid papillary carcinoma cell line TPC-1 [21] containing a CCDC6-RET fusion were used in this study. All cells were maintained in RPMI-1640 medium supplemented with 10% FBS, penicillin (100 U/mL), and streptomycin (10 μg/mL) in a humidified CO_2_ incubator at 37°C. All cells were passaged for less than 3 months before renewal from frozen early-passage stocks. Cells were regularly screened for mycoplasma using a MycoAlert Mycoplasma Detection Kit (Lonza, Rockland, ME, USA). Alectinib, vandetanib, and lenvatinib were obtained from Selleck Chemicals (Houston, TX, USA).

### Cell viability assay

Cell viability was measured using the MTT [3-(4,5-dimethylthiazol-2-yl)-2,5-diphenyl tetrazolium] dye reduction method. Tumor cells (2–3 × 10^3^ cells/100 μL/well) suspended in RPMI-1640 medium with 10% FBS were plated into 96-well plates and cultured with the indicated compound for 72 hours. Afterwards, 50 μg of the MTT solution (2 mg/mL, 21; Sigma, St. Louis, MO) was added to each well. Plates were incubated for 2 hours, the medium was removed, and dark blue crystals in each well were dissolved in 100 μL of DMSO. Absorbance was measured with a microplate reader at a test wavelength of 550 nm and a reference wavelength of 630 nm. Percent growth was determined relative to untreated controls. Experiments were repeated at least three times in triplicate.

### Antibodies and western blot analysis

Protein aliquots of 25 μg each were separated by SDS polyacrylamide gel electrophoresis (Bio-Rad, Hercules, CA) and transferred to polyvinylidene difluoride membranes (Bio-Rad). Membranes were washed three times and then incubated with Blocking One solution (Nacalai Tesque, Inc., Kyoto, Japan) for 1 h at room temperature. The membranes were incubated overnight at 4°C with primary antibodies against anti-RET (D3D8R) (#14698), anti-phospho-RET (Tyr905) (#3221), anti-AKT (#9272), anti-phospho-AKT (Ser473) (#4060), anti-cleaved PARP (#5625), anti-cleaved caspase-3 (#9664), and anti-β-actin (13E5) (#4970) antibodies (1:1,000 dilution each; Cell Signaling Technology, Danvers, MA). Additional antibodies were also used including anti-human/mouse/rat extracellular signal-regulated kinase (ERK) 1/ERK2 (0.2 μg/mL) (AF1576) and anti-phospho-ERK1/ERK2 (T202/Y204) (0.1 μg/mL) (AF1018) from R&D Systems. The membranes were washed three times and then incubated for 1 hour at room temperature with species-specific horseradish peroxidase-conjugated secondary antibodies. Immunoreactive bands were visualized with SuperSignal West Dura Extended Duration Substrate, an enhanced chemiluminescent substrate (Pierce Biotechnology, Rockford, IL). Each experiment was performed independently at least three times.

### Short interfering RNA knockdown

Duplexed Stealth RNAi (Invitrogen) against RET and a Stealth RNAi-negative control low GC Duplex #3 (Invitrogen) were used for RNA interference (RNAi) assays (DOC. S1). Briefly, aliquots of 1–2 × 10^5^ cells in 2 mL antibiotic-free medium were plated into each well of a 6-well plate and incubated at 37°C for 24 hours. The cells were transfected with siRNA (250 pmol) or scrambled RNA using Lipofectamine 2000 (5 μL) in accordance with the manufacturer’s instructions (Invitrogen).

### Tumor cell inoculation in immune-deficient mice

We used 6-week-old male SHO-Prkdc^scid^Hr^hr^ mice (SHO-SCID mice from Charles River, Yokohama, Japan) for the study. All animal experiments complied with the Guidelines of the Institute for Laboratory Animals of Advanced Science Research Center at Kanazawa University. For experiments using the pleural carcinomatosis mouse model [17], an incision was made in the skin and subcutaneous tissue on the right side of the chest to expose the parietal pleura. A 27-G needle was then used to inject tumor cells (1 × 10^6^/0.1 mL) through the parietal pleura into the right thoracic cavity. After inoculation, the quantity of tumors was tracked in live mice by repeated noninvasive optical imaging of tumor-specific luciferase activity using the IVIS Lumina XR Imaging System (PerkinElmer, Alameda, CA) [31] or CT scans. After sacrificing the mice, the volume of pleural effusion and weight of the thoracic tumor was evaluated, as described previously [32].

### Statistical analysis

Differences between groups were analyzed with one-way analysis of variance (ANOVA). All statistical analyses were performed using GraphPad StatMate 4 (GraphPad Software, Inc., San Diego, CA). *P* < 0.05 was considered significant.

## SUPPLEMENTARY MATERIALS FIGURES



## References

[1] Takahashi M, Ritz J, Cooper GM (1985). Activation of a novel human transforming gene, ret, by DNA rearrangement. Cell.

[2] Takahashi M (2001). The GDNF/RET signaling pathway and human diseases. Cytokine Growth Factor Rev.

[3] Prescott JD, Zeiger MA (2015). The RET oncogene in papillary thyroid carcinoma. Cancer.

[4] Tong Q, Xing S, Jhiang SM (1997). Leucine zipper-mediated dimerization is essential for the PTC1 oncogenic activity. J Biol Chem.

[5] Kohno T, Ichikawa H, Totoki Y, Yasuda K, Hiramoto M, Nammo T, Sakamoto H, Tsuta K, Furuta K, Shimada Y, Iwakawa R, Ogiwara H, Oike T (2012). KIF5B-RET fusions in lung adenocarcinoma. Nat Med.

[6] Takeuchi K, Soda M, Togashi Y, Suzuki R, Sakata S, Hatano S, Asaka R, Hamanaka W, Ninomiya H, Uehara H, Lim Choi Y, Satoh Y, Okumura S (2012). RET, ROS1 and ALK fusions in lung cancer. Nat Med.

[7] Lipson D, Capelletti M, Yelensky R, Otto G, Parker A, Jarosz M, Curran JA, Balasubramanian S, Bloom T, Brennan KW, Donahue A, Downing SR, Frampton GM (2012). Identification of new ALK and RET gene fusions from colorectal and lung cancer biopsies. Nat Med.

[8] Ju YS, Lee WC, Shin JY, Lee S, Bleazard T, Won JK, Kim YT, Kim JI, Kang JH, Seo JS (2012). A transforming KIF5B and RET gene fusion in lung adenocarcinoma revealed from whole-genome and transcriptome sequencing. Genome Res.

[9] Kohno T, Tsuta K, Tsuchihara K, Nakaoku T, Yoh K, Goto K (2013). RET fusion gene: translation to personalized lung cancer therapy. Cancer Sci.

[10] Drilon A, Rekhtman N, Arcila M, Wang L, Ni A, Albano M, Van Voorthuysen M, Somwar R, Smith RS, Montecalvo J, Plodkowski A, Ginsberg MS, Riely GJ (2016). Cabozantinib in patients with advanced RET-rearranged non-small-cell lung cancer: an open-label, single-centre, phase 2, single-arm trial. Lancet Oncol.

[11] Saito M, Ishigame T, Tsuta K, Kumamoto K, Imai T, Kohno T (2014). A mouse model of KIF5B-RET fusion-dependent lung tumorigenesis. Carcinogenesis.

[12] Huang Q, Schneeberger VE, Luetteke N, Jin C, Afzal R, Budzevich MM, Makanji RJ, Martinez GV, Shen T, Zhao L, Fung KM, Haura EB, Coppola D, Wu J (2016). Preclinical modeling of KIF5B-RET fusion lung adenocarcinoma. Mol Cancer Ther.

[13] Yoh K, Seto T, Satouchi M, Nishio M, Yamamoto N, Murakami H, Nogami N, Matsumoto S, Kohno T, Tsuta K, Tsuchihara K, Ishii G, Nomura S (2017). Vandetanib in patients with previously treated RET-rearranged advanced non-small-cell lung cancer (LURET): an open-label, multicentre phase 2 trial. Lancet Respir Med.

[14] Sakamoto H, Tsukaguchi T, Hiroshima S, Kodama T, Kobayashi T, Fukami TA, Oikawa N, Tsukuda T, Ishii N, Aoki Y (2011). CH5424802, a selective ALK inhibitor capable of blocking the resistant gatekeeper mutant. Cancer Cell.

[15] Kodama T, Tsukaguchi T, Satoh Y, Yoshida M, Watanabe Y, Kondoh O, Sakamoto H (2014). Alectinib shows potent antitumor activity against RET-rearranged non-small cell lung cancer. Mol Cancer Ther.

[16] Takeuchi S, Murayama T, Yoshimura K, Kawakami T, Takahara S, Imai Y, Kuribayashi Y, Nagase K, Goto K, Nishio M, Hasegawa Y, Satouchi M, Kiura K (2017). Phase I/II study of alectinib in lung cancer with RET fusion gene: study protocol. J Med Invest.

[17] Nakataki E, Yano S, Matsumori Y, Goto H, Kakiuchi S, Muguruma H, Bando Y, Uehara H, Hamada H, Kito K, Yokoyama A, Sone S (2006). Novel orthotopic implantation model of human malignant pleural mesothelioma (EHMES-10 cells) highly expressing vascular endothelial growth factor and its receptor. Cancer Sci.

[18] Ogino H, Yano S, Kakiuchi S, Yamada T, Ikuta K, Nakataki E, Goto H, Hanibuchi M, Nishioka Y, Ryan A, Sone S (2008). Novel dual targeting strategy with vandetanib induces tumor cell apoptosis and inhibits angiogenesis in malignant pleural mesothelioma cells expressing RET oncogenic rearrangement. Cancer Lett.

[19] Yokoyama A, Kohno N, Fujino S, Hamada H, Inoue Y, Fujioka S, Hiwada K (1994). Origin of heterogeneity of interleukin-6 (IL-6) levels in malignant pleural effusions. Oncol Rep.

[20] Suzuki M, Makinoshima H, Matsumoto S, Suzuki A, Mimaki S, Matsushima K, Yoh K, Goto K, Suzuki Y, Ishii G, Ochiai A, Tsuta K, Shibata T (2013). Identification of a lung adenocarcinoma cell line with CCDC6-RET fusion gene and the effect of RET inhibitors *in vitro* and *in vivo*. Cancer Sci.

[21] Tanaka J, Ogura T, Sato H, Hatano M (1987). Establishment and biological characterization of an *in vitro* human cytomegalovirus latency model. Virology.

[22] Rao PJ, Vardhini NV, Parvathi MV, Murthy PB, Sudhakar G (2014). Prevalence of RET/PTC1 and RET/PTC3 gene rearrangements in Chennai population and its correlation with clinical parameters. Tumour Biol.

[23] Ushenkova LN, Koterov AN, Biryukov AP (2015). Pooled Analysis of RET/PTC Gene rearrangement rate in sporadic and radiogenic thyroid papillary carcinoma. Radiats Biol Radioecol.

[24] Zhang T, Lu Y, Ye Q, Zhang M, Zheng L, Yin X, Gavine P, Sun Z, Ji Q, Zhu G, Su X (2015). An evaluation and recommendation of the optimal methodologies to detect RET gene rearrangements in papillary thyroid carcinoma. Genes Chromosomes Cancer.

[25] Wang R, Hu H, Pan Y, Li Y, Ye T, Li C, Luo X, Wang L, Li H, Zhang Y, Li F, Lu Y, Lu Q (2012). RET fusions define a unique molecular and clinicopathologic subtype of non-small-cell lung cancer. J Clin Oncol.

[26] Hechtman JF, Zehir A, Yaeger R, Wang L, Middha S, Zheng T, Hyman DM, Solit D, Arcila ME, Borsu L, Shia J, Vakiani E, Saltz L, Ladanyi M (2016). Identification of targetable kinase alterations in patients with colorectal carcinoma that are preferentially associated with wild-type RAS/RAF. Mol Cancer Res.

[27] Le Rolle AF, Klempner SJ, Garrett CR, Seery T, Sanford EM, Balasubramanian S, Ross JS, Stephens PJ, Miller VA, Ali SM, Chiu VK (2015). Identification and characterization of RET fusions in advanced colorectal cancer. Oncotarget.

[28] Wang K, Russell JS, McDermott JD, Elvin JA, Khaira D, Johnson A, Jennings TA, Ali SM, Murray M, Marshall C, Oldham DS, Washburn D, Wong SJ (2016). Profiling of 149 salivary duct carcinomas, carcinoma ex pleomorphic adenomas, and adenocarcinomas, not otherwise specified reveals actionable genomic alterations. Clin Cancer Res.

[29] Okamoto K, Kodama K, Takase K, Sugi NH, Yamamoto Y, Iwata M, Tsuruoka A (2013). Antitumor activities of the targeted multi-tyrosine kinase inhibitor lenvatinib (E7080) against RET gene fusion-driven tumor models. Cancer Lett.

[30] Taniguchi H, Takeuchi S, Fukuda K, Nakagawa T, Arai S, Yamada T, Yamaguchi H, Mukae H, Yano S (2017). Amphiregulin triggered EGFR activation confers crizotinib-resistance in a mouse model with EML4-ALK cancer and its circumvention with EGFR inhibitors. Cancer Sci.

[31] Nanjo S, Nakagawa T, Takeuchi S, Kita K, Fukuda K, Nakada M, Uehara H, Nishihara H, Hara E, Uramoto H, Tanaka F, Yano S (2015). *In vivo* imaging models of bone and brain metastases and pleural carcinomatosis with a novel human EML4-ALK lung cancer cell line. Cancer Sci.

[32] Li Q, Wang W, Machino Y, Yamada T, Kita K, Oshima M, Sekido Y, Tsuchiya M, Suzuki Y, Nan-Ya K, Iida S, Nakamura K, Iwakiri S (2015). Therapeutic activity of glycoengineered anti-GM2 antibodies against malignant pleural mesothelioma. Cancer Sci.

