# Supplementary material for: In vitro and in vivo anti-tumor activity of alectinib in tumor cells with NCOA4-RET
Source: Oncotarget. 2017 May 16;8(43):73766–73. doi: 10.18632/oncotarget.17900 (PMC5650298; doi:10.18632/oncotarget.17900)
Supplement: Supplementary file 1 [file oncotarget-08-73766-s001.pdf]

In vitro and *in vivo* anti-tumor activity of alectinib in tumor cells with NCOA4-RET

SUPPLEMENTARY MATERIALS

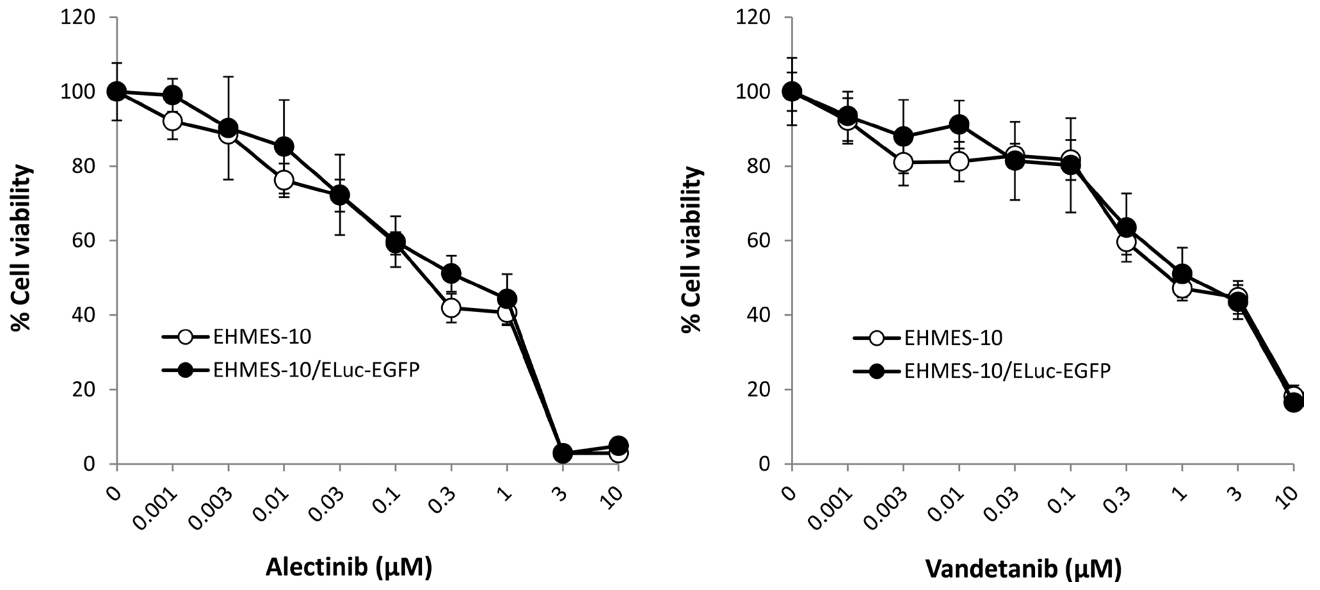

Supplementary Figure 1: EHMES-10/Eluc cells had similar sensitivities to alectinib and vandetanib compared to the parental EHMES-10 cells. EHMES-10 and EHMES-10/Eluc cells (2×10^3 cells/well) were incubated with various concentrations of alectinib or vandetanib for 72 hours. Cell viability was determined using the MTT assay. Bars represent SD of quadruplicate cultures. Data shown are representative of three independent experiments yielding similar results.

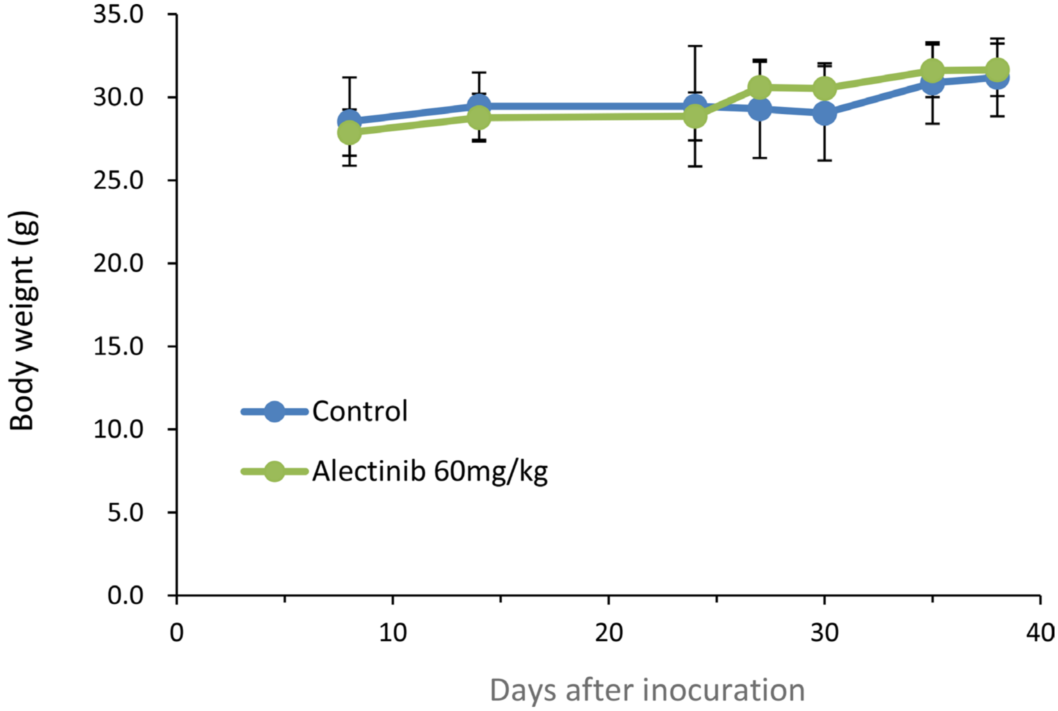

Supplementary Figure 2: Continuous treatment with alectinib at 60 mg/kg/day did not cause body weight loss of mice. The body weights of mice in Figure 5 were measured twice a week. Data shown are the means ± SD of five mice.

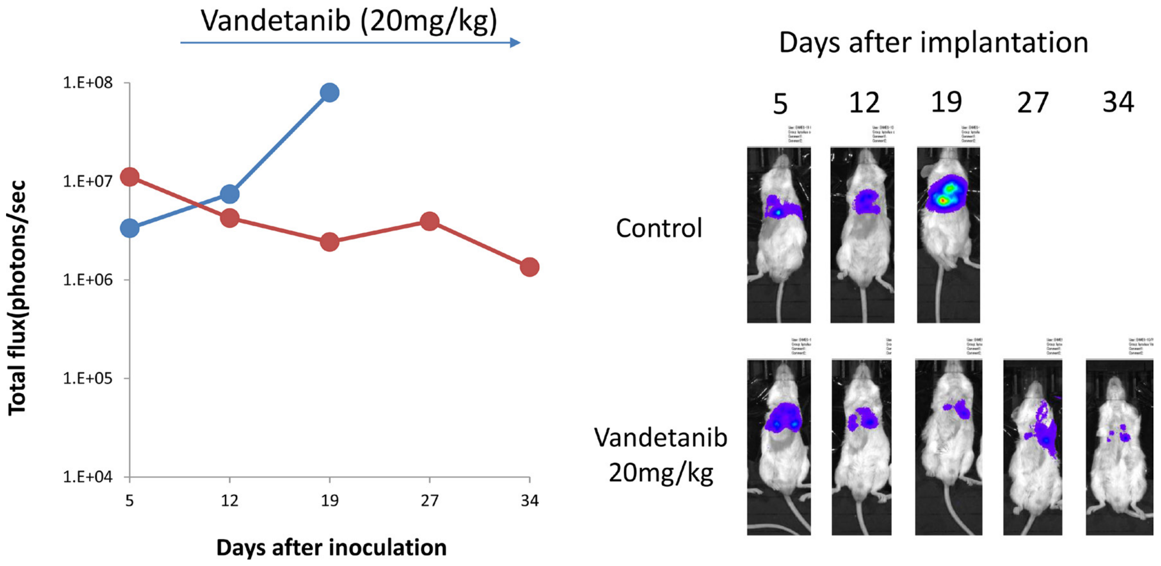

Supplementary Figure 3: Vandetanib inhibited the progression of EHMES-10/Eluc cells in the intrathoracic tumor model. A. EHMES-10/Eluc cells (1×10^6) were inoculated into the thoracic cavities of two SHO-SCID mice. Vandetanib treatment was given to one mouse from day 7 to day 34. Bioluminescence was measured by IVIS twice a week. B. Representative images are shown.
